# Double-hit lymphomas: clinical, morphological, immunohistochemical and cytogenetic study in a series of Brazilian patients with high-grade non-Hodgkin lymphoma

**DOI:** 10.1186/s13000-016-0593-0

**Published:** 2017-01-07

**Authors:** Cristiano Claudino Oliveira, Helena Maciel-Guerra, Luan Kucko, Eric Jun Hirama, Américo Delgado Brilhante, Francisco Carlos Quevedo, Isabela Werneck da Cunha, Fernando Augusto Soares, Ligia Niero-Melo, Patrícia Pintor dos Reis, Maria Aparecida Custodio Domingues

**Affiliations:** 1 Department of Pathology, Botucatu School of Medicine, São Paulo State University (FMB UNESP), Botucatu, São Paulo Brazil; 2 Botucatu School of Medicine, São Paulo State University (FMB UNESP), Botucatu, São Paulo Brazil; 3 Department of Pathology, AC Camargo Cancer Center, São Paulo, Brazil; 4 Department of Pathology, Amaral Carvalho Hospital, Jaú, São Paulo Brazil; 5 Department of Internal Medicine, Botucatu School of Medicine, São Paulo State University (FMB UNESP), Botucatu, São Paulo Brazil; 6 Departamento de Patologia, Faculdade de Medicina de Botucatu (FMB), Universidade Estadual Paulista (UNESP), Distrito de Rubião Junior, s/n°, Botucatu, SP Zip code: 18618-000 Brazil

**Keywords:** Lymphoma, Lymphoma, non-Hodgkin, Cytogenetics, *In situ* hybridization fluorescence, Immunohistochemistry

## Abstract

**Background:**

Double-hit lymphomas (DHL) are rare high-grade neoplasms characterized by two translocations: one involving the gene *MYC* and another involving genes *BCL2* or *BCL6*, whose diagnosis depends on cytogenetic examination. This research studied DHL and morphological and/or immunophenotypic factors associated with the detection of these translocations in a group of high-grade non-Hodgkin lymphoma cases.

**Method:**

Clinical and morphological reviews of 120 cases diagnosed with diffuse large B-cell lymphoma and Burkitt lymphoma were conducted. Immunohistochemistry (CD20, CD79a, PAX5, CD10, Bcl6, Bcl2, MUM1, TDT and Myc) and fluorescence *in situ* hybridization for detection of *MYC*, *BCL2* and *BCL6* gene translocations were performed in a tissue microarray platform.

**Results:**

Three cases of DHL were detected: two with translocations of *MYC* and *BCL2* and one with translocations of *MYC* and *BCL6*, all leading to death in less than six months. Among 90 cytogenetically evaluable biopsies, associations were determined between immunohistochemistry and fluorescence *in situ* hybridization for *MYC* (*p* = 0.036) and *BCL2* (*p* = 0.001). However, these showed only regular agreement, indicated by Kappa values of 0.23 [0.0;0.49] and 0.35 [0.13;0.56], respectively. “Starry sky” morphology was strongly associated with *MYC* positivity (*p* = 0.01). The detection of three cases of DHL, all resulting in death, confirms the rarity and aggressiveness of this neoplasm.

**Conclusions:**

The “starry sky” morphological pattern and immunohistochemical expression of Myc and Bcl2 represent possible selection factors for additional cytogenetic diagnostic testing.

## Background

Double-hit lymphomas (DHL) are aggressive neoplasms presenting translocation phenomenon involving the *MYC* gene combined with another event involving translocation of the *BCL2* or *BCL6* gene [1–6]. The combination of *MYC* translocation with translocations of *BCL2* and *BCL6* can also occur, defining the triple-hit lymphomas (THL) [1, 4, 7–9]. A diagnosis of DHL is only determined following the results of a cytogenetic test, such as fluorescence *in situ* hybridization (FISH) [10–12]. It represents approximately 15% of the provisional entity called B-cell lymphoma unclassifiable, with features intermediate between diffuse large B-cell lymphoma and Burkitt lymphoma (B-CLU/DLBCL/LB), Classification of Tumors of Haematopoietic and Lymphoid Tissues of the World Health Organization (WHO) in 2008 [1–4].

In 2016, Swerdlow et al. [13] published a proposal of revision in the WHO classification of 2008. This group of authors recognized DHL and THL as clinical and pathological categories, designated high-grade B-cell lymphomas (HGBL) with *MYC* and *BCL2* and/or *BCL6* rearrangements. Cases with morphological aspects of HGBL without *MYC* and *BCL2* and/or *BCL6* rearrangements were placed in the HGBL, NOS. However, there is not a specific guideline to select which cases should have FISH studies [13]. The morphological and/or immunophenotypic parameters do not seem to effectively predict the detection of cytogenetic translocations involving *MYC*, *BCL2* and *BCL6* [4, 14]. Molecular cytogenetics, like FISH, is not a readily available technique, mainly due to its high costs. Thus, determining which group of patients must have their biopsies submitted for this kind of test is a subject of controversy [4, 14, 15]. Despite the difficulties in interpreting the immunohistochemical profiles of Myc, Bcl2 and Bcl6, several studies have been published on how this technique can be used as a method of screening for molecular research, since it is being incorporated into the clinical routine as this cytogenetic information becomes increasingly relevant in therapeutic practice and prognosis [16, 17].

This study identified cases of DHL in a series of high-grade non-Hodgkin lymphomas in Brazil, characterizing them according to their clinical, morphological, immunophenotypic and molecular features. In addition, the associations between the expression of Myc, Bcl2 and Bcl6 proteins assessed by immunohistochemistry and the detection of translocations involving these genes were investigated in order to contribute to the discussions about how to select the cases of lymphoma for further molecular studies.

## Method

### Patients and study design

The authors selected 120 cases of patients with a pathological diagnosis of DLBCL or BL from two reference hospitals. Among the 120 cases selected following a review of pathology reports, 100 patients were diagnosed with DLBCL and 20 patients were diagnosed with LB. Patient records were reviewed from January 1998 to December 2013 at HC-FMB-UNESP, and from January 2010 to December 2011 at HAC. The study was approved by the Research Ethics Committee of FMB-UNESP, under protocol no. 4385–2012.

Sociodemographic data and clinical information were reviewed, including infectious comorbidities, history of non-Hodgkin lymphoma (NHL), presence of B symptoms, nodal and/or extranodal involvement, central nervous system (CNS) involvement, bone marrow infiltration (MO), levels of lactic dehydrogenase (LDH), Ann-Arbor staging, adopted therapeutic management, clinical and evolutionary tracking and recurrences.

### Morphological features

Hematoxylin and eosin (HE) slides of the 120 selected biopsies were evaluated according to the morphological criteria defined by the WHO, in its publication Classification of Tumours of Haematopoietic and Lymphoid Tissues, in 2008. The parameters adopted in this study were monomorphic/polymorphic cytological pattern, presence of “starry sky” pattern, diffuse growth, cytoplasmic eosinophilia, necrosis, mitotic activity, apoptosis, nuclear characteristics (vesicular, lobed, nucleolus, chromatin pattern), endothelial proliferation, stromal sclerosis and infiltration of adjacent soft tissues.

### Immunophenotypic evaluation, fluorescence *in situ* hybridization (FISH) and chromogenic *in situ* hybridization (CISH)

For immunophenotypic and cytogenetic evaluation, a tissue microarray platform (TMA) was constructed using cores from selected areas of each biopsy of the 120 cases. The 1.0 mm diameter cores were duplicated, and spaced every 0.2 mm [18].

Immunohistochemical analysis was performed using the polymer detection system with staining with diaminobenzidine chromogen and counterstaining with hematoxylin. The markers used were: CD20 (clone L26, dilution 1: 250, Cell Marque), CD79a (clone JCB117, Dako, ready to use), PAX5 (DAK-PAX5 clone, ready to use, Dako), CD10 (clone 56C6, ready to use, Dako), Bcl6 (PGB6-P clone, ready to use, Dako), Bcl2 (clone 124, ready to use, Dako), MUM1 (clone MUM1P, ready to use, Dako), TDT (polyclonal, ready to use, Dako) and Myc (clone EP121, ready to use, Biocare).

The expressions CD20, CD79a, PAX5, CD10, MUM1 and TDT were evaluated for positivity or negativity. The markers Myc, Bcl2 and Bcl6 were assessed for percentage of cellular expression. For Myc and BCL6, biopsies were considered positive when the marker expression was greater than 50% of tumor cells. For Bcl2, the criterion of positivity used was 70% or more cells stained by the marker. Evaluation of cell proliferation by Ki67 (Clone MIB-1, ready to use; Dako) was stratified into less than 90%, and equal to or greater than 90%. The authors classified the DLBCL cases with the Hans algotithm, determining immunohistochemical patterns compatible with the germinal center profile type (GCB) and the non-germinal center profile type, also named B cells activated (ABC).

Fluorescence *in situ* hybridization (FISH) analysis was performed on the cores contained in the TMA block using probes specific for translocation detection involving the loci of the *MYC* (Vysis LSI *MYC-IGH* dual color, orange and green, break apart rearrangement probe; Abbott Molecular Des Plaines, IL, USA), *BCL2* (Dako, *BCL2* FISH DNA, red and green, probe split signal) and *BCL6* genes (Vysis LSI, *BCL6*, dual color, orange and green, break apart probe rearrangement; Abbott Molecular Des Plaines, IL, USA), following the manufacturer’s instructions. The FISH results were analyzed by fluorescence microscopy (BX61; Olympus, Center Valley, PA) with appropriate filters and the images were captured with a Q-Color 5 Olympus digital camera. A case was considered positive when 5.0% of the cells (at least 40 tumor cells were counted) of a sample showed separate orange/red and green signals [19].

Chromogenic *in situ* hybridization (CISH) for EBV (Epstein Barr Virus) was performed on the cores of TMA block using Vortex ZytoFast CISH probe, following the manufacturer’s instructions. The CISH results were analyzed by optical microscopy, and each core was classified as positive or negative.

### Statistical analysis

All analyzes were performed using SPSS, version 22.0. The analysis was descriptive, followed by the Chi square test and Fisher’s exact test to compare proportions. The agreements between the immunohistochemical and FISH tests for the *MYC*, *BCL2* and *BCL6* markers were evaluated according to sensitivity, specificity, positive and negative predictive values, accuracy and Kappa coefficient, presented with their respective 95% confidence intervals. The strength of the agreement determined by the Kappa coefficient was analyzed as follows: poor (<0.20), regular (0.21 to 0.40), moderate (0.41 to 0.60), good (0.61 to 0, 80), very good (0.81 to 1.00). The significance level (*p*-value) tested was 0.05.

## Results

The 120 patients were submitted for clinical review, morphological analysis, immunohistochemistry and molecular evaluation. However, in 30 cases, flaws were verified in molecular testing in one or more of the three markers, related to issues of material fixation. FISH analysis detected three cases of DHL. The three groups defined by the cytogenetics investigation were BL (*n* = 15), DHL (*n* = 3) and DLBCL (*n* = 72); the sociodemographic, clinical, morphological, immunohistochemical and cytogenetic data are shown in Table 1.

**Table 1:** Sociodemographic. clinical. morphological. immunohistochemical and cytogenetic data of the diagnostic groups after FISH

	Diagnostic Groups after FISH					
	BL (*n* = 15)		DHL (*n* = 3)		DLBCL (*n* = 72)	
	n	%	n	%	n	%
Sociodemographic data						
Woman	6	40	2	66.7	29	40.3
Man	9	60	1	33.3	43	59.7
Age over 60 years old	0	0	3	100	34	47.2
HIV	1	6.7	0	0	2	2.8
LNH history	0	0	0	0	2	2.8
Viral hepatitis	0	0	0	0	4	5.5
Morphology						
Monomorphic	15	100	3	100	51	70.8
Polymorphic	0	0	0	0	21	29.1
“Starry sky” pattern	14	93.3	0	0	17	23.6
Diffuse growth	15	100	3	100	71	98.6
Necrosis	1	6.7	1	33.3	26	36.1
Mitotic activity	12	80	1	33.3	52	72.2
Apoptosis	14	93.3	3	100	57	79.1
Vesicular nuclear pattern	4	26.7	1	33.3	52	72.2
Evident nucleolus	5	33.3	0	0	64	88.8
Lobed nucleous	2	13.3	0	0	30	41.6
Dense chromatin	10	66.7	3	100	18	25
Loose chromatin	6	40	0	0	54	75
Endothelial proliferation	4	26.7	1	33.3	35	48.6
Cytoplasmic eosinophilia	7	46.7	3	100	43	59.7
Soft tissue infiltration	4	26.7	1	33.3	38	52.7
Stromal sclerosis	0	0	0	0	19	26.3
Immunohistochemistry						
CD20	15	100	3	100	68/70	97.1
CD79a	14	93.3	2	66.7	66/70	94.3
PAX5	11	73.3	1	33.3	49/70	70
CD10	15	100	3	100	31/71	43.6
Bcl6	7	46.7	2	100	22/70	31.4
Bcl2	0	0	2	100	29/71	40.8
MUM1	0	0	0	0	37/71	52.1
TDT	0	0	0	0	0	0
Myc	5	33.3	0	0	11/70	15.7
Ki-67 > 90%	3	20	2	66.7	9/70	12.8
Cytogenetic						
*MYC*	15	100	3	100	3	4.2
*BCL2*	0	0	2	66.7	11	15.3
*BCL6*	0	0	1	33.3	9	12.5
Prognostic markers						
High levels of LDH	5/6	83.3	2	66.7	37/47	78.7
Staging III/IV	9	60	0	0	29/71	40.8
Bone marrow infiltration	2	13.3	0	0	12/71	16.9
CNS infiltration	1	6.7	0	0	4/71	5.6
B symptoms	9	60	1	33.3	31/71	43.6
Extranodal involvement	8	53.3	2	66.7	35/71	49.3
Nodal involvement	8	53.3	1	33.3	44/71	61.9
Therapeutic data						
Chemotherapy	11	73.3	2	66.7	65/71	91.5
Chemotherapy and radiotherapy	1	6.6	0	0	17	26.6
Bone marrow transplant	0	0	0	0	1	1.4
Death	5	33.3	3	100	30/66	45.4
Recurrence	0	0	0	0	2/59	3.4

### Clinical aspects

Patients with BL were predominantly male (60%), with a median age of four years old (range: 2–48 years old), and included one with HIV. These patients exhibited elevated LDH level (83.3%), stage III/IV in clinical presentation (60%), extranodal masses (83.3%) and lymphadenopathy (53.3%). Among patients with BL, five patients (33.3%) died, approximately 1.2 months after diagnosis (SD = 0.83).

Among patients with DLBCL, males predominated (59.7%) and the median age was 56.5 years old (range: 25–69 years old), with 47.2% of patients over 60 years old. Extranodal masses and enlarged lymph nodes were present in 49.3 and 61.9% of cases, respectively. Regarding treatment, 57 (79.2%) of the 72 cases diagnosed with DLBCL were submitted to the CHOP (cyclophosphamide, doxorubicin hydrocloryde, vincristine sulfate, prednisone) regimen, 11 were submitted to a combination of CHOP with rituximab (R-CHOP) and 14 required other therapeutic regimens. As for clinical outcomes in the DLBCL group, 30 of the 72 cases (41.7%) resulted in death about 13 months after diagnosis (SD = 13.99).

The three cases of DHL (Table 2) were two women and a man, all over 60 years old, who presented no comorbidities. One of this group was previously diagnosed as BL; the others, as DLBCL. None of them received a B-CLU/DLBCL/LB diagnosis. Clinically, the initial presentation in two patients was extranodal mass. B symptoms and enlarged lymph nodes were described in one patient. Neoplastic infiltration was not detected in the bone marrow or CNS in all three patients. None of these cases presented Ann-Arbor stage III or IV. The clinical course of the DHL patients was death in one, five and six months, regardless of chemotherapy. One individual was prescribed the R-CHOP regimen and another, the R-HIPERCVAD regimen (cyclophosphamide, vincristine, doxorubicin and dexamethasone). The patient that died in less than 30 days did not start chemotherapy.

**Table 2:** Clinical, immunophenotypic and cytogenetic of the three patients with double-hit lymphomas (DHL)

	Patient 1	Patient 2	Patient 3
Age	65 year old	72 year old	64 year old
Gender	Woman	man	woman
Previous diagnosis	BL	DLBCL	DLBCL
Immunohistochemistry			
CD20	positive	positive	positive
CD79a	negative	positive	positive
PAX5	positive	negative	positive
CD10	positive	positive	positive
Bcl2	negative	positive	positive
Bcl6	positive	negative	positive
Myc	negative	negative	negative
MUM1	negative	negative	negative
TDT	negative	negative	negative
Ki-67	>90%	>90%	<90%
Cytogenetic			
*MYC*	positive	positive	positive
*BCL2*	negative	positive	positive
*BCL6*	positive	negative	negative
Clinical data			
Staging	IA	IA	IB
Levels of LDH	High	ND*	ND*
Bone marrow infiltration	absent	absent	absent
CNS infiltration	absent	absent	absent
Extra nodal involvement	absent	presence	presence
Nodal involvement	presence	absent	absent
B symptoms	absent	absent	presence
Treatment	HIPER-CVAD	Not treated	R-CHOP
Death	Yes	yes	Yes
Interval to death	5 months	1 month	6 months

*ND=not avaible

### Morphological aspects

The monomorphic and diffuse pattern with a “starry sky” appearance was the main aspects of the BL group, as expected according to the literature. Among the DLBCL patients, the morphological pattern of diffuse growth was frequent (98.6%), with a “starry sky” pattern in 23.6% of the cases studied.

In the DHL group, the typical morphology in our study was diffuse monomorphic pattern, eosinophilic cytoplasm and apoptotic elements. The “starry sky” pattern, sclerosis or nuclear lobulation were not observed in this group.

### Immunohistochemical aspects

The B immunophenotype was confirmed in all cases of BL. Positivity for CD10 (100%) and Bcl6 (46.7%) were also observed.

In the DLBCL group, among 70 patients, 34 were GCB type and 36 were ABC type. For the ABC type patients, in the immunohistochemistry analysis, one was BCL6 positive, 20 were Bcl2 positive and two patients are positive for Myc and Bcl2.

The three DHL had a B immunophenotype. MUM-1, TDT and MYC were negative in all three biopsies. One case was positive for both BCL6 and BCL2. Cell proliferation, measured by Ki-67, was higher than 90% in two cases.

### Molecular aspects

Molecular cytogenetic by FISH showed that all 15 patients diagnosed as BL presented translocation involving the *MYC* gene. In this group, one of five patients over 18 years old tested positive for EBV on the CISH test.

In the DLBCL group, positivity for *MYC*, *BCL2* and *BCL6* was determined in three (4.2%), nine (12.5%) and 11 cases (15.3%), respectively. In these patients, a statistically significant association (*p* = 0.011) between the positivity for *MYC* translocation and the “starry sky” morphological pattern was noted. Concerning EBV status, the CISH revealed ten positive cases in the DLBCL group.

**Fig. 1:**
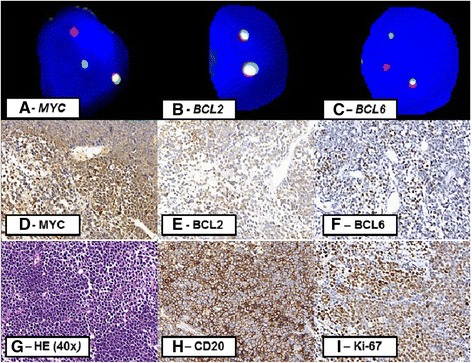
Morphological, cytogenetic and immunohistochemical aspects of patient 1. Key: Images **a**, **b** and **c** correspond to FISH: there is positivity for *MYC* (**a**) and *BCL6* (**c**), in this case, the signals are indicated with arrows, and there is negativity for *BCL2* (**b**). Images **d** (400x) and **e** (400x) exhibit the negativity for Bcl2 (below 70% of marked cells) and Myc in immunohistochemistry (below 50% of marked cells). In image **f** (400x), there is BCL6 positivity (above 50% of marked cells). Image **g** (H&E, 400x): the diffuse pattern in morphology. Positivity for CD20 confirms immunophenotype B and is shown in image **h** (400x). Image **i** (400x) refers to cell proliferation rate above 90%

One of the three DHL patients presented translocation of the *MYC* gene simultaneously with the *BCL6* gene translocation (Patient 1; Fig. 1) and the others presented translocation involving the *MYC* gene simultaneously with the *BCL2* gene (Patients 2 and 3). In this series, there were no cases of triple-hit lymphoma. None of the three patients tested positive for the EBV infection on CISH test.

Correspondence between the cytogenetic findings and immunohistochemical profile for the entire set of cases is shown in Table 3. Immunohistochemical positivity for Myc was verified in 19 cases, eight (42.1%) of which were also positive in the cytogenetic analysis (*p* = 0.036). For this marker, the immunohistochemical technique showed high specificity (85%) and low sensitivity (38%), with regular agreement at identifying the respective translocation (Kappa = 0.23 [0.0,0.49]). Regarding Bcl2, the most interesting aspect is the high NPV, which is 95% (*p* = 0.001), with regular agreement (Kappa = 0.35 [0.13;0.56]). Four (12.5%) of the 32 cases positive for Bcl6 by immunohistochemistry showed positivity in FISH (*p* = 0.724), indicating poor agreement between the two methods for this marker (Kappa = 0.04 [0.00;0.30]). The findings related to *BCL6* gene were not statistically significant.

**Table 3:** Immunohistochemistry (IHC) for Myc, Bcl2 and Bcl6 and cytogenetic (FISH) for *MYC*, *BCL2* and *BCL6*, considering the total number of valid samples: sensitivity (S), specificity (E), positive predictive value (PPV) and negative predictive value (NPV), accuracy (A) and Kappa coefficient, with respective ranges of 95% confidence

	n	FISH+	p*	S	E	PPV	NPV	A	Kappa
MYC (n = 92)									
IHC negative	73	13 (18.0%)	0.036	0.38	0.85	0.42	0.82	0.74 [0.65;0.83]	0.23 [0.0;0.49]
IHC positive	19	8 (42.1%)							
BCL2 (n = 101)									
IHC negative	64	3 (4.6%)	0.001	0.81	0.71	0.35	0.95	0.73 [0.64;0.81]	0.35 [0.13;0.56]
IHC positive	37	13 (35.1%)							
BCL6 (n = 98)									
IHC negative	66	6 (9.0%)	0.724	0.40	0.68	0.12	0.90	0.65 [0.56;0.75]	0.04 [0.0;0.30]
IHC positive	32	4 (12.5%)							

*p < 0.05

Evaluation of these associations considering only patients with DLBCL are shown in Table 4. In this group, nine cases (31%, *p* = 0.005) showed immunohistochemical and cytogenetic positivity for *BCL2*, with a regular agreement (Kappa = 0.29 [0.04; 0.54]). Parameters, such as a cell proliferation rate above 90%, an ABC type, and immunohistochemical positivity for Myc, were not associated with the detection of *MYC* translocation by FISH.

**Table 4:** Immunohistochemistry (IHC) for Myc, Bcl2 and Bcl6 and cytogenetic (FISH) for *MYC*, *BCL2* and *BCL6*, considering the group of DLBCL: sensitivity (S), specificity (E), positive predictive value (PPV) and negative predictive value (NPV), accuracy (A) and Kappa coefficient, with respective ranges of 95% confidence

	n	FISH+	p*	S	E	PPV	NPV	A	Kappa
MYC (*n* = 70)									
IHC negative	59	1 (1.7%)	0.062	0.67	0.87	0.18	0.98	0.86 [0.78;0.94]	0.23 [0.0;0.67]
IHC positive	11	2 (18.1%)							
BCL2 (*n* = 71)									
IHC negative	42	2 (4.7%)	0.005	0.81	0.66	0.31	0.95	0.69 [0.58;0.79]	0.29 [0.04;0.54]
IHC positive	29	9 (31%)							
BCL6 (*n* = 70)									
IHC negative	48	6 (12.5%)	1.000	0.33	0.69	0.13	0.87	0.64 [0.53;0.76]	0.01 [0.0;0.32]
IHC positive	22	3 (13.6%)							

*p < 0.05

## Discussion

DHL are rare, aggressive neoplasms that account for less than 10% of B-cell lymphomas and about 4% of high-grade lymphomas [4, 12]. This first Brazilian study found three cases of DHL, two with translocations involving *MYC* and *BCL2*, and one with translocations involving *MYC* and *BCL6*. The combination that is most often reported is the double translocation involving the *MYC* gene and the *BCL2* gene (62%) [4, 20].

The most important clinical and prognostic factors of DHL patients are the advanced stage at diagnosis, the presence of extranodal masses, the bone marrow or CNS infiltration and the age of over 60 years old [4, 10, 12, 16, 21]. The three patients identified in this study were elderly; however, they did not present high Ann-Arbour stages or bone marrow/CNS infiltration, though two of the patients exhibited extranodal masses.

Survival is lower in patients with DHL. In this study, all three patients with DHL died, an average of 4 months after diagnosis. According to the literature [16], the overall median survival rate is 8.2 months in this group of patients *versus* 56.8 months in patients with other high-grade lymphomas. Snurdel et al. (2010) [10] reported 70% of deaths within eight months in patients with DHL. The aggressiveness of DHL is possibly explained by the synergistic action of *MYC* and *BCL2*: proliferative activity with inhibition of apoptosis in the context of a complex karyotype [4, 20, 22, 23].

In this study, the best agreements between immunohistochemical and FISH results occurred with respect to the *MYC* gene and, mainly, the *BCL2* gene, with high NPV. Evaluations of the *MYC* gene and the *BCL2* gene shows good results, however, there is a fair agreement by Kappa coefficient, indicating the need for caution in assessing the markers with the immunohistochemical method.

Immunohistochemical evaluation of Myc, Bcl2 and Bcl6 is a problem discussed by several hematopathology groups [12, 22–24]. The cutoff for determining positivity for MYC varies between 30 and 50% in the literature, while for Bcl2, the variation is between 30 and 70% [22, 23, 27].

Perry et al. [24] studied the immunohistochemical expression of Myc and Bcl2 of 106 patients diagnosed with DLBCL, correlating their data with prognosis. Positivity for these markers, especially when both were positive, was related to worse prognosis, increasing the chances of death nine-fold. However, Perry et al. (2014) did not correlate their results with cytogenetic data [24].

Green et al. [25] studied the immunohistochemical and cytogenetic profiles of 193 patients diagnosed with DLBCL, of which 6% were DHL identified by FISH, and showed poor prognosis. Regarding immunohistochemistry, 29% of patients were positive for Myc and Bcl2, and 54% of these patients had translocations involving one of these genes. These patients, regardless of the cytogenetic test results, showed worse survival rates compared with other patients [25].

Yan et al. [26] evaluated 336 patients with DLBCL by immunohistochemistry and FISH, and found a high specificity (approximately 90%) of positivity for Myc and Bcl2 in the immunohistochemistry in relation to FISH positivity for *MYC* and *BCL2* translocations, favoring its potential use for stratification in cases of DLBCL with aggressive behavior [26]. Kawamoto et al. [15] studied only DLBCL patients, by FISH and immunohistochemistry. They found that *MYC* translocation and Bcl2 immunohistochemical expression were independent prognostic factors and must be investigated at the initial diagnosis [15].

Kalaw et al. [28] studied the cell proliferation rate, measured by the percentage of staining for Ki67, as a potential selection parameter of patients for cytogenetic tests. The cutoff values used were 75% or 90% and both were not associated with detection of *MYC* translocations, as was seen in this study.

Mahmound et al. (2015) [27] studied the agreement in the evaluation of Myc expression by immunohistochemistry and noted great heterogeneity in the results, reflecting the subjective interference and the lack of a clear standard regarding the interpretation of this marker [27].

In this study, the “starry sky” morphological pattern, in the DLBCL context, positive immunohistochemistry for Myc in over 50% of cells, and positive immunohistochemistry for Bcl2 above 70% are indicated as potential selector parameters for patients eligible for cytogenetic study. However, further studies with larger case samples are required, for more detailed analysis of these associations, in special morphological pattern studies because it is a conventional and accessible method of evaluation in the pathology routine.

Swerdlow (2014) [23] emphasizes the importance of identifying cases of aggressive large B cell lymphomas, mainly due to the absence of a specific therapeutic protocol [23]. In this context, there is great interest in studies of agreement between immunohistochemical and cytogenetic results, mainly in relation to selector elements for molecular screening. In fact, difficulties related to the standardization of defining criteria of positivity for Myc and Bcl2 in immunohistochemical reactions has been reported.

## Conclusions

This study presents the first Brazilian series of patients with double-hit lymphoma identified by cytogenetic study performed by FISH on 120 cases of high-grade lymphomas. For this study, high grade B-cell lymphomas, particularly when there is a “starry sky” pattern, must be submitted to immunohistochemistry for, at least, Myc andBcl2. When Myc and/or Bcl2 are positive, if it is possible, the investigation must continue with FISH test for *MYC*, *BCL2* and *BCL6*. These cases are high grade lymphomas and the molecular markers must be reported. In the situation that molecular approach is not possible, the immunohistochemistry results must be reported too, because they may represent a prognostic factor.

## Abbreviation

ABC: B cell activated or non-germinal centre
B-CLU/DLBCL/LB: B-cell lymphoma unclassifiable, with features intermediate between diffuse large B-cell lymphoma and Burkitt lymphoma
BL: Burkitt lymphoma
CISH for EBV: Chromogenic *in situ* hybridization (CISH) for EBV (Epstein Barr Virus)
CNS: Central nervous system
DHL: Double-hit lymphomas
DLBCL: Diffuse large B-cell lymphoma
Fish: Fluorescence *in situ* hybridization
GCB: B germinal centre
HE: Hematoxylin and eosin
LDH: Lactic dehydrogenase
MO: Bone marrow infiltration
NHL: Non-Hodgkin lymphoma
THL: Triple-hit lymphomas
TMA: Tissue microarray platform
